# Motivations for volunteering time with older adults: A qualitative study

**DOI:** 10.1371/journal.pone.0232718

**Published:** 2020-05-04

**Authors:** Anne Same, Hannah McBride, Caitlin Liddelow, Barbara Mullan, Courtenay Harris

**Affiliations:** 1 School of Occupational Therapy, Social Work, and Speech Pathology, Faculty of Health Sciences, Curtin University, Perth, Western Australia, Australia; 2 School of Psychology, Faculty of Health Sciences, Curtin University, Perth, Western Australia, Australia; University of Indianapolis, UNITED STATES

## Abstract

Improved health, wellbeing and quality of life are associated with older adults living well at home. Enabling older adults to remain at home requires organisations to consider different workforce models to support these initiatives. Volunteers are often used by organisations providing such services. However, given the changing nature of the volunteer industry, volunteer recruitment and retention practices must be better understood. This study sought to understand individuals’ motivations to volunteer in aged care home support. Eighteen volunteers were recruited from not-for-profit aged support organisations in Perth, Western Australia. Semi-structured interviews were conducted, and five themes were thematically interpreted: What’s Important to Me?, Learning and Growth, Somewhere to Belong, Meet Me in the Middle, and Not Just a Number. Fulfilling volunteers’ desires for interest, social connection, self-growth, recognition, and support appeared conducive to positive volunteering experiences. These findings may suggest avenues to capture and retain volunteers in aged care home support.

## Introduction

As of 2017, there were approximately 3.8 million (15% of total population) Australians over the age of 65 years [1]. It is expected that the number of older Australians will continue to grow, with an estimated 8.8 million (22% of total population) older adults by 2057 [1]. With the growing number of older adults comes an increasing demand for quality aged care services. However, the aged care sector in Australia has struggled to both attract and retain workers due to low pay rates and a lack of secure employment opportunities [2]. In an attempt to reduce the stress on residential care facilities, the Australian government created the Commonwealth Home Support Programme (CHSP) which aims to keep older adults living independently at home and in the community for longer [3]. During the 2017–2018 financial year, 1,456 not-for-profit organisations providing at home support services were funded by the CHSP [4]. In 2016, it was estimated that approximately 23,000 Australians volunteered their time in residential aged care facilities in a two-week period and approximately 44,000 volunteered in home care and support in the same time period [5]. Official reports show a slight increase in volunteer numbers in residential care per fortnight (1,276 more volunteers) from 2012 to 2016. Conversely, there has been a decrease in both the number of volunteers per fortnight (14, 850 less volunteers) and the overall number of hours volunteered per fortnight (51, 842 less hours) in home care and support services from 2012 to 2016 [5].

Recent changes in social context, such as longer working hours, dual-income households, and reduced opportunities for holidays [6,7] has made engagement in volunteering less feasible for the majority of people. As of 2014, it was estimated that 31% of Australian adults engaged in formal voluntary work within a structured organisation, a slight drop from 2010 when approximately 34% of Australian adults volunteered [8]. Previous research has shown particular groups within the community are more likely to volunteer compared to others. For example, females are more likely to volunteer compared to males [8] and similarly those with a bachelor’s degree or higher are more likely to engage in voluntary activities than those without such qualifications. Although understanding which groups in society are more likely to engage in volunteering is beneficial, it does not always aid in successful recruitment and long-term retention of volunteers. Research suggests that motivations are the most important factor in both the initiation and maintenance of volunteering behaviour [9–12].

The most common approach to exploring the motivations of volunteers is the functionalist approach [13]. The functionalist approach of volunteer motivations [14,15] was developed using quantitative methods, and focuses on both the social and personal functions of volunteering behaviour, which are contributed to by the individual’s thoughts, feelings and actions. The underlying assumption of the theory is that people volunteer in order to fulfil underlying social and psychological functions [14]. Prior research has found that people volunteering in similar roles may have different underlying motivations for engagement. For instance, across a range of studies involving volunteers from health, social outreach, disaster relief, and university settings, Clary and colleagues [15] identified several key motivations underlying volunteering; protective, values, career, social, understanding and enhancement. Protective motivations are described as those which protect the ego from negatively perceived aspects of the self, such as by reducing guilt experienced via realising others’ disadvantage [15]. Values-oriented motivations are present where a person volunteers as a vehicle for expressing or enacting their own personal values, often aligned with benefitting others, or altruism [15]. People may also volunteer due to career-related motivations, where individuals perceive volunteering as beneficial in gaining industry-specific experiences, skills, or contacts [15]. Social motives may be served by volunteering, in that individuals may have the opportunity to spend time with others or build new relationships and social networks [15]. Volunteering may also be motivated by a desire to engage with new experiences, learning, or skills-building, as described by the Clary and colleagues’ understanding function [15]. Finally, enhancement motivations encapsulate those wherein a person volunteers due to self-oriented reasons, such as self-development or self-esteem [15].

Following on from this research, Stukas and colleagues [16] found that sporting volunteers reported their primary motivation to be either social engagement, or enhancement and self-growth. Thus, understanding individual motives for voluntary engagement is important for both initiating and maintaining volunteers. Volunteering motives have primarily been explored using quantitative methods, such as with Clary and colleagues’ research [see 9,16–19]. However, some qualitative studies have successfully identified and developed different typologies and theoretical insights by interviewing volunteers [20,21]. Previous qualitative studies have suggested that further qualitative research with volunteers is needed to identify motives not otherwise captured using quantitative methods [22], and thus build on the existing literature on volunteer motivations.

Although studies have previously identified motives across various volunteering sectors [see 22–24], research exploring the motives for volunteering in aged care organisations is limited. Existing research in aged care volunteering primarily focusses on the psychological and physical benefits for older adults who volunteer [25–27], or the experiences of older adult volunteers who engage in both informal and formal volunteering [28,29], rather than the reasons individuals volunteer in the aged care sector.

### The current study

With an Australian Government initiative geared toward keeping older adults in their homes for longer, but with a decreasing number of volunteers and total volunteer hours in this area, the need to both identify and understand why volunteers in this sector are motivated to volunteer is important. To guide this research, the overarching research question was: “what motivates individuals to volunteer their time, and continue to volunteer time, for organisations providing aged care home support services?”. The aim of this study was to qualitatively explore the underlying motivations of volunteers who are currently, or have previously volunteered, for an organisation providing aged care home support services.

## Method

### Overview

This research used a qualitative research design, wherein semi-structured interviews were employed to gather contextually rich data from interviewees. Volunteer interviewees were recruited through social media advertisements, contact with volunteer agencies and via snowball sampling by explicitly asking interviewees if any of their friends or colleagues came to mind for participation in the study. Researchers adhered to methodological guidelines by Braun and Clarke [30] throughout the collection of qualitative data and thematic analysis of interview transcripts. Ethical approval was obtained from the University’s Human Research Ethics Committee (HRE2017-0861).

### Participants

The researchers conducted semi-structured interviews with a total of 18 participants, with both current volunteers (*N* = 11) and recently exited volunteers (*N* = 7) from a Perth aged care home support service organisation. A total of 7 males and 11 females were interviewed, ranging from 23–79 years of age, with one month of volunteering experience to 16 years with their organisation. Almost all interviewees were living in the Perth Metropolitan area, with one participant from regional Western Australia. Twelve interviewees were retired, three unemployed but seeking work, one unemployed under other circumstances (e.g. medical), one was both working and studying, and another was working full-time. Current and previous volunteers had primarily volunteered in such settings as gardening, home maintenance, at-home help services, transport, office settings, and shopping and other outings. Participants had the choice to be interviewed either in person or over the phone. To be eligible for the study interviewees needed to be currently residing in Western Australia, over the age of 18 years, and engaged in a current volunteer role or have recently exited a volunteering role in an aged care home support service.

### Interview schedule and procedure

A semi-structured interview guide consisting of 14 questions was used to obtain qualitative, contextually rich data from interviewees. The guide was developed through collaboration with psychologists and occupational therapists with prior experience in studies on the topic of volunteering. To best investigate diverse factors around volunteering, the questionnaire aimed to explore three primary areas; volunteer motives, volunteer experiences, and volunteer thoughts and appraisals of volunteering, with example questions including “What were your motivations or reasons for volunteering?”, “Why did you choose this service e.g. gardening, transportation, home care?”, and “Can you tell me about why you chose this organisation to volunteer with?”.

It has previously been suggested that data saturation can occur within a minimum of 12 interviews [31], but that heterogeneity among the sample can influence this number. Given this, 15 interviews were conducted over the phone, and three face to face. After obtaining informed consent either digitally or in-person, researchers asked volunteers questions from the semi-structured interview guide, utilising prompts and additional questions to explore points of personal relevance and interest to each interviewee.

With consent to participate given orally, interviews were recorded by the interviewer and later transcribed verbatim in preparation for analysis. Interviews ran for approximately 35–45 minutes each. Any names or other identifying information were redacted from transcripts throughout the transcription process and pseudonyms have been used throughout.

### Data analysis

Volunteer interviews were thematically analysed by researchers two and three using an inductive thematic analysis approach, following the six guidelines proposed by Braun and Clarke [30]. An inductive thematic analysis is where the analysis is grounded in the data rather than from existing theories. Each transcript was approached with a preliminary read-through for researcher familiarisation with the data by two researchers. Both researchers then interpreted analytic codes, line-by-line, from the transcribed interviews. These codes were further refined into latent codes, and then into themes and subthemes to better capture the underlying ideas of each dataset. This process was repeated for each transcript until no further themes could be interpreted from the data, and thematic saturation was reached [32]. Throughout analysis, researchers conducted some coding independently and some in collaborative meetings, in the interest of researcher triangulation. If disagreements arose during the process of theme identification, they were resolved through collaborative discussion between researchers. Once consensus had been reached regarding the identification and segmentation of themes, themes were collaboratively named and defined, and further reviewed by the rest of the team. Themes were sent to other team members for review and feedback. This was done to strengthen dependability by ensuring no radical interpretations, and to reduce potential researcher bias. Relevant quotes were selected from a diverse range of the analysed transcripts to add further depth to the themes interpreted from the data [30].

### Quality procedures

To ensure quality and rigour in the current study as well as to allow triangulation, a team of researchers divided the transcripts for analysis and met multiple times throughout this process. The engagement in such confirmability processes aimed to reduce any subjectivity or bias in the researcher’s interpretations and analysis of the data, increasing trustworthiness [33]. To increase dependability, the researchers involved in data collection participated in reflexive journaling, after each interview, to reflect on their own social position and how potential personal biases may influence the research process [34]. Additionally, the researchers involved in data analysis journaled any broader ideas or concepts which appeared pertinent in relation to underlying themes throughout the data. Although the sample was from one not-for-profit organisation in a singular city, which may limit generalisability, the diverse age range, occupation and time engaged in volunteering allows for findings to be generalisable to wider populations.

## Findings

The analysis revealed that volunteers in one Perth aged care home support service organisation expressed motives that were found to align with five key themes: What’s Important to Me?, Learning and Growth, Somewhere to Belong, Meet Me in the Middle, and Not Just a Number.

### What’s important to me?

This theme encompasses the importance of personal interest and role enjoyment expressed by interviewees when speaking about their volunteering experiences in aged care. Many interviewees mentioned that their current volunteer roles encompassed tasks they either enjoyed or were initially interested in partaking in. For instance, Nancy was involved in a garden maintenance role and said, “I love gardening and I love talking to people and that seemed a good fit …” and another, Karen, commented “I was trying to retire and do something interesting …”. Through this commentary, it appears that interviewees considered their own interests in deciding whether to volunteer, and in guiding the nature of the volunteer role they might engage in, such as garden maintenance, driving or assisting with shopping. More simply, these factors appeared to not only motivate interviewees to volunteer, but also to help them determine the role they choose.

Interviewees also noted that variety in volunteer roles and settings was a way to keep their volunteering experiences dynamic and engaging. For instance, Muhammad said “…we’ve even discussed you know, like every fortnight we go to (*organisation name*) or something like that and then for the other weeks, we go to a different organisation and do something else. Just to give that sort of difference…”. Here, the interviewee outlines the importance of variety, and their willingness to seek it out where not provided by their current organisation. It appears that being presented with a variety of different roles or tasks may create the perception of a more dynamic environment with which volunteers can engage, and thus create interest.

Many interviewees also spoke to the importance of engaging in a volunteering role aligned with their values. Volunteers appeared to express a sense of satisfaction from their volunteer work where their tasks contributed to a cause which held some personal value for them. For example, Tao and Daniel stated “I’ve always wanted to give back to the community. You know?”, and other interviewees, Michael, Suzanne, Louise, and Cheng, stating, “And we were doing something good to help other people…”. It seems that in these two quotes, values of altruism and wanting to give back to the community in a selfless way were a key motivator to volunteer. Knowing that being in this volunteering role was benefitting the health and independence of older adults appeared to be important to the volunteers.

In some interviews, volunteers mentioned changes in their mental health. When recalling the time around their mother’s death, one interviewee, Victor, said “…that sort of put a big black cloud over my head. I think that doing what I’m doing now has really helped in that aspect as well.”. Here Victor refers to their experiences with volunteering as having had a positive impact on their life, in the face of an adverse life event. Although perhaps not an initial motivator, it may be that the impact of volunteering on positive mental health outcomes is one realised by volunteers, and encourages continuation in volunteer roles.

#### Finding purpose

Finding Purpose describes participants’ aspirations to meaningfully engage in everyday life. For some interviewees, finding a volunteer position seemed to be underpinned by a desire to find a role to keep themselves busy, or to stay active and out in the community—especially in the absence of formal employment or in cases of retirement. For example, Karen said “…I decided that I wanted to retire and close up my business–everything and I needed something to do.”, and Daniel noted “…I don’t feel like I’m just doing nothing. Even though maybe I’m not doing that much. It’s better than doing nothing …”. It seems that for some, a lack of activity is perhaps synonymous with a sense of purposelessness. Volunteering may be a way of bringing about a sense of achievement or direction in everyday life, remaining interested and engaged, and is perhaps especially valued by those whose schedules have slowed down.

### Learning and growth

This speaks to interviewees’ positive appraisals of engaging in volunteer environments and learning new skills through their experiences. Across interviewees, these instances of personal growth were cited as being role-related, interpersonal, or to do with emotional self-growth. Overall, having opportunities to expand skillsets or capabilities—whether conducive to employment or to everyday life—seemed to be an attractive element of the volunteer experience for those interviewed. For example, Tao who is in a gardening and home maintenance role commented “…it teaches me things that I can use myself here on my own property.”, and Cheng, who is in a similar role, noted “I got a certificate to use a chainsaw and quite a few other things. Which benefitted me.”. In these excerpts, interviewees highlighted the value of building on their existing repertoire of skills to be applied not only in their role, but also in other areas of their lives.

Other interviewees, however, appeared to take more value-laden lessons away from the volunteer experience. In their interview, Daniel said, “I’ve learnt that I can do more than I thought I could.” and also stated “I’m not someone that can just walk up to someone and start talking to them and I think the social interaction … with the volunteering, it’s actually given me a new lease on life.”. In these instances, interviewees speak to a greater sense of confidence and self-efficacy, which they seem to attribute to having gained as part of their experiences in aged care volunteering and engagement with a diverse range of community members.

Some participants also mentioned that they believed learning new skills and gaining experience through volunteering could facilitate future employment opportunities. This can be seen when Suzanne said “…it can also open up doors for employment later on. If you have liked worked long enough and the general going out there and meeting people and working with a team …”. Here, the interviewee describes a skillset of networking and teamwork as potentially helpful in seeking future employment.

### Somewhere to belong

Interviewees expressed that opportunities to meet others, engage socially, and become part of a community were important elements of the volunteer experience. When discussing some initial reasons for volunteering, Nancy said “I also wanted to participate in the community was the main reason. Just be part of the community I lived in and to help people.”. Whilst for Nancy it seems like finding a broader sense of community was an initial motive, for others it appeared to be a potentially unexpected yet enjoyable part of their volunteer role and subsequently the volunteer continuing their role. This can be seen when Suzanne, Muhammad, Tao and, Daniel noted similar experiences “I’m starting to go to social. I would recommend it because if they loved going out and meeting new people or doing stuff or if they just want to like join a group, it’s nice to have people that you can talk to.”.

It was noted by some interviewees that volunteering could be an opportunity in which to meet others with similar interests. One interviewee, Charlie, highlighted their positive social experiences through volunteering where they noted “I do like the volunteering thing and it’s kind of nice to get out and it helps to meet people and like-minded people.”. Thus, some participants may perceive volunteering as a positive environment in which to foster meaningful connections with others, built on a foundation of commonality and comradery. Interviewees also spoke positively of group social events planned by their organisation. This is seen, for instance, when Maria recalled “…all the depots used to get together, and we’d have a central breakfast in a big hall or something. Then it got down to–we’d just have a get-together in May of every depot.”. On finding a common time to suit many volunteers in a large organisation, Victor noted “Even if it’s only an hour or one day a month. The hardest thing is trying to get everybody together. But I don’t think that should stop it happening.”. Although Victor appears to empathise with difficulties in planning larger events, having access to social opportunities is still valued and is perhaps a criterion for many volunteers when selecting an organisation to volunteer under. Where an organisation seeks to plan these social opportunities, it may serve to foster an organisational ethos which mirrors volunteers’ own values of teamwork and community. Thus, in instances where an organisation makes the effort to plan social events, volunteers may feel as though their organisation hears their feedback and understands what is important to them.

### Meet me in the middle

The accessibility of volunteering settings and opportunities was interpreted as a salient topic when discussing factors conducive to a positive volunteer experience with interviewees. Some participants highlighted that having close volunteering sites made volunteering more feasible, subsequently influencing their willingness to continue in their role. For instance, Nancy said “…it’s sort of ‘local’ to me as well, the depot for gardening. I can walk there and also I can run if I need to get there quickly…”. This excerpt appears to perhaps highlight Nancy’s willingness to engage on an ad-hoc basis, or fit volunteering in with other components of a busy lifestyle, in the instance they might need to travel quickly to site. However, on discussing what changes to location might mean for volunteers, Victor responded “…if they moved from (*one suburb*) to (*another suburb*) or somewhere. You know, that’s time to get there and whereas it’s convenient for me to volunteer where I’m volunteering now”. A volunteer, Lee, who was interviewed shortly after resigning from their volunteering role said:

*The only challenge that I had personally was because I don’t drive*, *I found it a bit of challenge to get from where I’m living to the venue—to get from the venue and to get home again*. *Because although it’s on bus route*, *it’s in not a very nice area*.

Thus, it may be that interviewees perceive volunteering as more viable if conveniently located, and less viable if not. Similarly, feelings of safety also appear to be important such that if the role is located in an area with an unsafe reputation, volunteers may be deterred from engaging within that role.

Commentary from some interviewees also pointed to the importance of volunteering fitting in with existing aspects of their lives. For instance, Nancy and Victor expressed “…they’re flexible. Like I didn’t have to commit to being there every week or something. Like I’ve got other things that I do. I can’t always do it because I’ve got grandkids” and “…I do like the flexibility of the place. If I want to go to Bali for a month–as long as I tell them in advance, there’s no problem”. Although cited as a positive activity to engage in, volunteering may not be a top priority for all people, and as such the flexibility of any role is an important motivation for both initial engagement and continued engagement. Volunteering appears to be viewed as more feasible when not impinging on other areas of participants’ lives, offering volunteers the opportunity to volunteer when it is suitable for them.

### Not just a number

Volunteers expressed the need to feel supported and heard by their organisation, and having their efforts acknowledged by both the organisation and the people for whom they volunteer. For instance, one volunteer, Tao, said, “The supervision we get from the organisation is good. The co-ordinators that we work with are very good. Yeah. It’s good in that way.”, and Charlie commented “They’re always asking you ‘Are you sure you can do that’ or you know, ‘Do you want a hand with that or’–now they’re always very protective of their volunteers.”. In these quotes, interviewees seem to express an overall sense of support from the on-site guidance they are afforded by their organisation and as such, creating a sense of comfort.

In other excerpts, volunteers spoke to specific instances of personalised support received from their organisation. In an instance where one volunteer, Michael, was having difficulty getting to site, their support worker noted, “She spoke to her manager and they organised for the HACC taxi to actually bring (*name redacted*).”. In arranging transport for Michael to get to site, the organisation appeared to address the volunteer’s specific needs, and facilitate their engagement in volunteering. Actions like these may ultimately help volunteers feel heard and supported by their supervising organisation, especially in instances where volunteers are experiencing personal difficulties affecting volunteering.

Communication was also highlighted by volunteers as an important factor in feeling supported. For instance, on modifying their organisation’s existing methods of communication, one interviewee, Muhammad, said:

…*just even a text like on the day*. *Like not me just having to call up*. *If they’d just say ‘Oh*, *you know*,*’ because literally all I’d do is say ‘What run are we going to be on tomorrow*?*’ and that’s it*. *Like they could literally just send me a text and then I’d know because I spoke to them*.

Effective methods of communication between co-ordinators and volunteers may facilitate current volunteers continuing their volunteer role, especially where they feel their needs are recognised as important by the organisation.

Instances of recognition from the organisation and customers alike were spoken to as enjoyable aspects of interviewees’ volunteer experiences. Regarding recognition from their organisation, Michael mentioned “They celebrate the volunteers and they have volunteer awards.”, and Victor noted “They will have a Christmas picnic. All those little things to say ‘thank you’ and it’s something that is really good and to know that you are appreciated. You know, from the organisation.”. Further, Victor, speaking to expressions of appreciation from customers cited “…that does make you feel really good. When they appreciate what you do.” and Tao said “…volunteering is giving your time when you don’t get paid. You know? No, I enjoy it and we have support, because the volunteers are appreciated *(by customers)*.” In these excerpts, appreciation or recognition of effort appears to be conducive to volunteers appraising their experiences as positive. Thus, recognition is perhaps an important part of a positive volunteer experience and may encourage volunteers to continue to contribute their time and energy to these organisations, knowing they are not only helping those who need it, but that recipients of services provided are appreciative of volunteers’ hard work and time.

## Discussion

The purpose of this study was to explore the motivations of individuals who volunteer their time with aged care home support services. The findings of the present study indicate there are a wide variety of motivations that underlie initial engagement in aged care home support volunteering roles, as well as the continued engagement of volunteers in this setting. It appears these findings align with some of Clary and Snyder’s [15] volunteer functions, such as ‘values’, ‘understanding’, ‘enhancement’, and ‘social’ motives. Although conceptualised as a single overarching theme in this analysis, paradigms of skill acquisition and personal development captured by the theme ‘Learning and Growth’ appear consistent with Clary and Snyder’s [15] functions of 'understanding’, where one might seek to develop knowledge related to new skills, and ‘enhancement’, which describes personal and psychological growth. Similarly, topics of personal importance addressed in the theme ‘What’s Important to Me?’ appear to share characteristics with the function of ‘values’ posited by Clary and Snyder [15]. In addition, parallels can be drawn between the theme ‘Somewhere to Belong’, and Clary and Snyder’s [15] ‘social’ function, both of which highlight the importance of building social connections and finding a sense of community.

Despite these similarities, the present research differentiates itself from Clary and Snyder’s [15] theory by identifying important external motives for volunteering. Such examples include the theme ‘Meet Me in the Middle’, regarding the accessibility of nearby volunteering opportunities, and the importance of organisation and consumer recognition described by ‘Not Just a Number’. These themes both appear to describe factors which are external to the volunteer; although expressed in terms of volunteer need, these themes describe elements of the volunteering experience which can be determined by the organisation through culture and policy. Primary differences in the findings of the present study and the functionalist approach could perhaps be attributed to a cultural shift; since the creation and refinement of the functionalist approach, the needs and attitudes of volunteers may have shifted, as well as the demands of the settings, roles, and organisations with which volunteers are now engaging.

The motivations found by the present study appear to align more with egoistic motives, rather than altruistic attitudes. This finding is not uncommon in the literature, with previous research also identifying egoistic motives for volunteering engagement being more pertinent than altruistic motives [35,36]. Specifically, volunteers may now hold preconceived expectations around receiving something in return for their efforts [37]. Altruistic motivations may bear relevance where an individual holds such humanitarian values as improving the welfare or livelihood of others, with no anticipated gain of their own; the theme ‘What’s Important to Me?’ may capture some aspects of altruism, dependent on the individual’s own values and interests. However, the primary themes interpreted in the present research perhaps sway toward egoism, where volunteers were seen to express desire for or satisfaction of fulfilment in such areas as social engagement, skills building, and fostering interest. This dichotomy between altruistic and egoistic motives provides an avenue for future research in aged care home support volunteers, such that further understanding of the underlying attitudes may provide implications for changes in volunteering policy and marketing.

Research by Son and Wilson [38] posited that increases in self-esteem encouraged volunteers to remain in their position for a longer stretch of time. This notion was echoed in the present research in the theme Learning and Growth, such that it appears that the positive impacts of volunteering on self-efficacy and self-confidence are recognised by volunteers as valuable by-products of the volunteering experience. Similarly, elements of social connectedness and community discussed in the theme Somewhere to Belong have been highlighted as salient factors in volunteers staying in roles over time [38,39]. The notion of volunteers being recognised as individuals by their overarching organisation, as discussed in the theme Not Just a Number, is supported by Phillips and Phillips [35] whose research suggested that opportunities for communication from a diverse range of the organisation’s volunteers could help individuals feel heard by their organisation.

Further, the findings of this study, when considered within a broader context of the existing research into volunteer motives, may ultimately suggest that the motivations of volunteers across diverse volunteering settings may be underpinned by similar foundational principles. Given the diversity of volunteering settings (e.g. sporting, environmental, disability, aged home care), it may simply be that finding ways to fulfil volunteers’ motivations and needs must be adapted across different contexts [40].

### Implications

Across aged care home support volunteering contexts, volunteers may be more attracted to organisations offering a range of roles, such as outdoor volunteer positions in gardening and transport, or indoor roles like shopping assistance or general support. Thus, organisations should promote the choice of roles they can offer to prospective volunteers. Similarly, aged care home support services should consider highlighting in marketing material how volunteering provides benefits, in attempts to appeal to the egoistic motives expressed by a number of their volunteers. Advertisements that attempt to appeal to egoistic motives (e.g. advertising “get your foot in the door”) may be a viable option. Organisations may also consider meeting with current volunteers to ensure their volunteering goals and motivations are being fulfilled by the organisation, such as CV-building or social engagement. Such strategies may aid in volunteer retention in ensuring a mutually beneficial relationship between volunteer and organisation. It may be that policy makers and volunteering organisations consider providing more opportunities for volunteers to benefit themselves, as purely altruistic behaviour seems to be less important in today’s busy and time-poor society [35,36].

To further both recruitment and retention of egoistically motivated volunteers, organisations might invest in upskilling volunteers in a range of areas useful to both the role and the individual outside of their volunteering position. Advertising the availability of workshops or certificates, such as first aid, manual handling courses, specialised driving for transport volunteers, or training in use of equipment, may show volunteers and prospective volunteers that the organisation is willing to invest in and support them, whilst also providing a two-way benefit between individual and organisation.

The findings also suggest that opportunities for individuals to engage in large-scale social events with other volunteers are an important part of the volunteering experience. It may be that such events foster greater feelings of community and belonging for volunteers and could perhaps build a stronger volunteer network. Organisations should consider arranging regular social events, which may encourage existing volunteers to stay involved with the organisation long term, knowing they are able to engage socially with a community of like-minded individuals. Similarly, in the interest of retention, recognition of volunteer efforts through certificates, awards ceremonies, ‘employee of the month’ nominations, and other such incentives may create a culture where volunteers feel appreciated, and further strive to perform, potentially at little cost to the organisation and its consumers. Finally, none of these recommendations are achievable without organisations taking the time to consider the unique perspectives of their volunteer workforce. Opening channels for feedback and communication may address volunteers’ desires to feel heard, whilst also cultivating a deeper understanding of what volunteers within a specific setting may need.

### Strengths and limitations

The present study is the first study which has both endeavoured to understand the motivations of individuals volunteering in aged care home support, as well as explore these motivations qualitatively and inductively. As a methodological strength of the study, both current and recently exited volunteers were interviewed to understand a range of individual perspectives. However, as the study was conducted with one organisation in Perth, Western Australia, the findings may not be widely generalisable, and should be interpreted with this in mind. Additionally, member checking was not conducted following data collection or analysis, which may reduce the trustworthiness or credibility of findings. In future qualitative research into volunteer motivations, employing member checking processes would strengthen methodological quality. A final limitation of this study may be the presence of bias in some interviews, as volunteers who had recently left the organisation may have reported poorer experiences, and those still within the organisation may have spoken more positively about their volunteering. To mitigate this, the findings were critically evaluated by two researchers familiar with the datasets, to ensure a breadth of experiential perspectives were captured, as well as the broader themes underlying those perspectives. Future research should consider investigating the motives of volunteers in aged care home support organisations in areas outside of Perth, as well as other aged care volunteer settings, such as retirement villages or support work. This would help expand an understanding of volunteer motivations across the aged care sector.

## Conclusion

In the context of an ageing population and healthcare systems unequipped to support that growth, the present research sought to understand motivations underlying volunteers in aged care home support settings, in the interest of beginning to discern what might be needed to grow an efficient and viable volunteer workforce. Interviews with current or recently exited volunteers identified five primary areas of importance for volunteering: What’s Important to Me?, Learning and Growth, Somewhere to Belong, Meet Me in the Middle, and Not Just a Number. In instances where some or all of these needs were cited as being met, interviewees seemed to speak more positively to experiences of volunteering, and noted intentions to either continue volunteering, or re-engage if they had recently left a role. Conversely, those who reported negative outcomes across these five broader areas appeared to express less satisfaction and willingness to continue in their position. It is imperative to find ways to successfully recruit and retain volunteers in aged home support contexts, in the interest of developing a strong and skilled volunteer work force in this area. Through this, the volunteer industry can effectively support ageing in place by improving the health, wellbeing and quality of life of older adults, whilst simultaneously reducing burden on existing healthcare systems.

## Supporting information

S1 Data (PDF)
