# Supplementary material for: Motivations for volunteering time with older adults: A qualitative study
Source: PLoS One. 2020 May 4;15(5):e0232718. doi: 10.1371/journal.pone.0232718 (PMC7197773; doi:10.1371/journal.pone.0232718)
Supplement: S1 Data — (PDF) [file pone.0232718.s001.pdf]

Part 1 - Latent codes

Current Volunteers - Latent Codes

Participant 1 - Maria

- Time to give
- interest
- social outlet
- staying active
- need to fit needs
- interest
- personal growth
- comfortable role
- personal growth
- fulfilment
- family encouragement
- skills building
- interest
- support from organisation
- comfortable with staff
- open communication
- supported by org
- valued by org
- (looked after?)
- volunteering isn't work
- comradery
- community
- comradery
- valued work
- fulfilling
- social engagement
- interests
- part of something
- achievement
- social
- values social outlet
- community
- social outlet
- social aspect is important
- fulfilment
- valued
- social outlet
- recognition = important

Participant 2 - Tao

- work alternative
- convenient
- giving back
- accessible info
- work alternative
- interest
- fit with life/needs
- interest
- more casual than work
- personal interest
- interest
- empathy
- (personal connection to clients)
- social outlet
- personal connections (e.g. friendships, closeness)
- community
- personal connections
- variety = interesting
- personal connections
- social outlet
- community
- staying active
- personal benefits
- skills building
- personal gain
- knowledge seeking
- empathy
- build own understanding
- required to volunteer
- staying active
- personal gain
- valued
- interest
- fits with life
- fit with needs/preferences
- low commitment
- convenient
- volunteering isn’t work
- social
- community

Participant 3 - Muhammed

- helping others
- fulfilment

- personal growth
- helping
- fits with life
- needs to fit with schedule
- related to existing skills
- (familiar work)
- social engagement
- need to fit life
- need to fit life
- personal growth
- social outlet
- personal growth
- positive impact for clients and volunteers
- social outlet
- personal growth
- personal growth
- fulfilment
- older people volunteer
- retirement
- convenience
- convenient
- convenience
- convenient
- support
- community
- family support
- interest
- fulfilling
- social aspect
- community
- personal growth
- fulfilment
- achievement

Participant 4 - Nancy

- *volunteering for social reasons -> more friendship than professionalism*
- something to do
- keeping busy
- value for other people
- community integration
- social engagement
- accessible
- easy application - facilitated
- personal values
- aligned with interests

- staying active
- social engagement
- easy tasks
- personal interest
- belonging
- support
- interest in variety
- engaged interests
- connection with clients
- fulfilling
- health benefits
- active
- aligned with values
- health benefits
- personal gain
- applying existing skills
- fulfilment
- social engagement
- part of community
- convenient
- fits with life
- fits with schedule
- more casual than work
- comradery
- low stress
- not work
- good deed
- low stress
- convenient
- fits with life

Participant 5 - Suzanne

- social engagement
- ease of application
- ease of access/application
- positive volunteer environment
- support
- value/acceptance
- personal interest
- aligned with values
- prosocial action
- personal growth
- personal growth
- personal growth
- supported by org

- interests/needs supported
- social engagement
- personal growth
- creates opportunity
- personal growth
- gain positive attributes
- positive volunteer environment
- community
- supported by organisation
- values support
- positive volunteer environment
- support from others
- community
- positive community = important
- support
- positive community
- personal growth
- social engagement
- community
- volunteering = social?
- social growth
- positive social experiences
- value
- appreciation
- areas of interest
- social engagement
- community
- valued by organisation
- value organisation listening
- support from organisation
- prosocial/altruistic
- fulfilment

Participant 6 - Michael

- social encouragers
- fulfilment
- social encouragement
- social encouragement (friends)
- staying engaged
- keeping busy
- valued
- keeping busy/engaged
- social encouragement
- accessibility
- dynamic role

- engaging/enjoyable
- fulfilment
- prosocial action
- interests facilitated
- enjoyment
- self-improvement (mental health)
- positive volunteer environment
- positive volunteer environment
- self-improvement
- positive volunteer environment
- social opportunities
- positive volunteering environment
- community
- positive volunteering environ
- appreciation
- support
- support
- positive volunteer environment
- community
- support
- support (family, volunteers, organisation)
- barriers to volunteering
- support from org
- support from org
- personalised support
- support from org
- appreciation
- value
- supported
- unexpected benefits
- helping others
- prosocial action
- personal gain
- self-improvement
- community
- supported
- personal gain
- fulfilment
- barrier (less autonomy) - values autonomy
- personal gain
- positive impact on life
- personal drive
- supported to continue role
- individualised support
- values good communication

- supported by org
- consistency in role
- not as serious as work
- teamwork
- fits with life
- convenient
- social opportunities
- appreciated
- valued
- social opportunities
- personal growth
- community
- social opportunities

Participant 7 - Daniel

- poor life circumstances
- interest
- altruism
- prosocial action
- personal growth
- stepping stone to employment
- social engagement
- social encouragement
- learning
- facilitate positive change
- personal investment
- family encouragement
- improved QOL
- employment opportunities/raises employability
- personal growth
- prosocial action
- valued
- help needy
- fulfilment
- support from org
- social encouragement
- seeing others fulfilled
- fulfilling
- personal growth
- mutual benefit
- supportive environment
- personal growth
- self-worth
- fulfilment
- accomplishment

- self-esteem/worth
- social engagement
- positive social experiences
- volunteering is better than nothing - something to do?
- learning
- focus on others
- personal investment
- care
- convenience
- organisation listens
- support between staff - comradery
- support from training
- care for clients
- support from staff
- comradery
- barriers to helping
- uncertainty
- value for clients
- appreciates support/guidance
- appreciates guidance
- client-centred work
- guidance
- care for clients
- personal growth
- fulfilment
- helping needy
- prosocial action
- role variety
- socialising
- positive volunteer environment
- socialising
- positive volunteer environment
- client-focussed
- support valued
- client-focussed
- support
- client-focus
- care
- learning via experience
- equipping to help clients
- views organisation positively
- mutual benefits

Participant 8 - Victor

- poor previous role

- convenient
- accessible
- desire for change
- social encouragement
- convenience
- fits lifestyle
- convenience
- accessibility
- supportive/positive environment
- supportive management
- role diversity
- personal interest
- personal interest
- variety
- social engagement
- building bonds
- enjoying new experiences
- choice of roles
- aligned with values (e.g. active)
- fits with life
- role variation
- fits with life
- convenient
- listened to by org
- personal growth
- change from work
- fulfilment from others' happiness
- client focus
- positive environment
- support system
- social encouragement
- fits with life
- recognition
- feels valued
- personal growth
- empathy for clients
- personal investment/connection
- care/empathy
- personal fulfilment
- prosocial action
- valued
- feels useful
- learning
- helping others
- client autonomy

- personal growth
- empathy
- client-focus
- positive environment
- support in training
- support from org/management
- appreciation/valued
- organisation hears volunteers
- input valued
- able to offer skills/insight
- felt listened to by org
- valued
- adequate training
- forgotten
- value congruence between intention/action
- feel valued
- supported by org
- autonomy
- responsibility
- personal growth
- helping those in need is fulfilling
- appreciation
- recognition of sacrifice
- feel valued
- fulfilling
- rewarding
- empathising
- personal understanding
- positive volunteer environment
- social encouragement
- awareness of services
- social encouragement from others
- values enough to encouraging others
- fit with lifestyle
- personal benefit/gain
- fits with life
- convenient
- fulfilment
- sees volunteering as part of lifestyle
- diverse roles to suit needs
- support to continue
- convenient
- low effort
- accessible
- fits with life

- values communication
- volunteer communication
- comradery
- mutual learning
- communication
- communication is important
- importance of being heard
- recognition of effort
- valued by organisation
- clear client appreciation
- less clear appreciation from org
- focus shifted from volunteer recognition
- expect appreciation
- social encouragement
- comfortability
- values social opportunities
- organisation can facilitate community
- social engagement
- client-focussed
- care for clients
- uncomfortable tasks
- heard by organisation
- valued/listened to
- values good management/coordination
- values punctuality
- organisation/time-management
- volunteer time not valued
- frustrated
- supported by org
- community
- fulfilment
- fits with life
- org. values time given
- appreciation
- fits with life

Participant 9 - Charlie

- job alternative
- enjoyment
- convenient
- easier than job-seeking
- valued by others
- recognition
- accessibility
- personal interest

- active interests
- values
- devalued due to age
- social engagement
- personal growth
- purpose
- improved mindset/ QOL
- perseverance
- positive QOL/outcomes
- personal understanding
- social engagement
- challenging
- volunteering = extra
- supportive org
- enjoys volunteering more than work
- easier than working
- value autonomy
- org supports volunteers
- communication with org
- values communication
- support from org
- expected easier role
- pension is motivation - convenient
- reduction of stress
- volunteering seen as easy
- social encouragement
- expected volunteering easier
- life uncertainty
- volunteering is an extra
- not a priority
- fits with life
- social engagement
- personal focus
- clients value service
- accessible
- volunteering - preference due to low stress
- volunteering seen as low stress
- volunteering seems less about values and more about personal benefit for this volunteer. Own financial focus, prioritising own financial circumstances, doesn't seem as motivated. Less language about clients and some apprehension around role. Volunteering to alleviate financial stress - seen as easy solution, and then realised it isn't so easy. Overarching idea that volunteering should be easy. Is volunteering categorised with recreation rather than work?

Participant 10 - Louise

- job opportunities
- employability
- values opportunity
- reputable organisation
- diverse roles
- personal growth
- skills building
- personal interests
- willing to contribute lots of time
- organisation is equitable
- makes good/fair decisions for volunteers
- supportive org
- personal interest
- valued service
- positive view of org
- aligned with values
- prosocial action
- experience for employment
- personal gain
- social engagement
- community
- personal growth
- self worth
- personal gain
- engaging
- supportive organisation
- positive view of org
- personal gain
- growth
- supported by org
- values communication
- support
- positive environment
- supportive organisation
- approachable
- clear communication
- opportunity
- own skills valued
- supportive org
- opportunities
- valued in role
- valuable work/contribution
- variety in tasks
- belonging
- contribution valued

- worthwhile
- growth
- achievement
- community
- org doing good for volunteers
- mutually beneficial
- accepting and supportive environment

Participant 11 - Karen

- personal interest
- alternative to work
- spare time used productive
- volunteer in absence of work
- personal interest
- prosocial/altruistic
- fulfilment
- volunteering isn't work
- altruism
- help needy if can
- improve work ethic
- UNFINISHED TRANSCRIPT

Unsure if relevant (maybe not **motivation**)

- personal connection to clients
- longstanding connections
- structure restricts connection
- organisational change = changed focus
- personal connection with clients
- clients vs friends
- volunteers previously more valued
- growth = disconnect
- not heard by org
- not valued throughout merger
- value from org = important
- no volunteer recognition
- not heard by org
- communication
- expect org to listen
- communication
- input
- communication
- more structure than expected
- more structure than expected

- expected more casual
- age
- support from organisation
- change for better/worse?
- VTF different to other organisations
- communication
- support from organisation
- adequate training
- hands on learning
- more learning
- supported by organisation
- sacrifice time
- age-related barriers
- prefer less structure
- client focussed work
- personalised
- more social
- company listens to needs/wants
- positive view of org
- client-focussed
- communication
- difficulty accessing
- communication
- communication breakdowns
- communication
- communication
- lost interest in role
- change if not suitable
- change if unsuitable
- personalised care
- more convenience
- barriers to application
- org. flexibility re change
- two way communication
- positive volunteer environment
- positive impact on mentality
- change, uncertainty
- organisational support
- client centred care
- communication
- barriers to care
- procedural barriers
- genuine bonds
- change
- restricted by rules

- client focussed care
- communication
- communication
- restricted by rules
- clients or closer
- genuine bonds
- friendship
- good support = personalised
- genuine bonds
- perseverance
- organisation appreciation

Part 2 - Reorganisation of themes into codes

Current Volunteers - Codes to themes

- interest
- interest
- interest
- interest
- interests/needs supported
- personal interest
- interests facilitated
- enjoyment
- aligned with values
- areas of interest
- interest
- variety = interesting
- interest
- personal values
- aligned with interests
- interest in variety
- personal interest
- engaged interests
- aligned with values
- interest
- interest
- personal interest
- interests
- role diversity
- personal interest
- personal interest
- variety
- interest
- enjoying new experiences
- personal interest
- active interests
- enjoyment
- personal interests
- engaging
- personal interest
- values
- personal interest
- personal interest
- aligned with values (e.g. active)
- personal investment

- staying active
- something to do
- keeping busy
- staying active
- staying engaged
- keeping busy
- staying active
- health benefits
- active
- staying active
- keeping busy/engaged
- volunteering is better than nothing - something to do?
- health benefits
- spare time used productively
-

- fulfilling
- fulfilment
- fulfilment
- fulfilling
- fulfilment
- fulfilling
- fulfilment
- fulfilment
- fulfilment
- fulfilment
- valued work
- achievement
- achievement
- helping
- valued
- value/appreciation
- valued
- recognition = important
- value
- appreciation
- fulfilment
- fulfilment
- valued
- appreciation
- value
- appreciated
- appreciation

- valued
- seeing others fulfilled
- altruism
- valued
- fulfilment
- fulfilling
- fulfilment
- accomplishment
- fulfilment
- fulfilment
- appreciation
- recognition of sacrifice
- feel valued
- fulfilling
- rewarding
- fulfilment from others' happiness
- valued
- feels useful
- valued
- recognition
- feels valued
- fulfilment
- appreciation
- fulfilment
- appreciation/valued
- valued by others
- recognition
- purpose
- feel valued
- recognition of effort
- valued in role
- contribution valued
- achievement
- valuable work/contribution
- valued service
- own skills valued
- fulfilment
- personal fulfilment
- valued by org /(looked after?)

- personal growth
- skills building
- personal benefits
- personal gain

- skills building
- personal gain
- personal growth
- personal growth
- personal growth
- personal growth
- gain positive attributes
- personal growth
- personal growth
- personal growth
- personal growth
- personal growth
- personal growth
- personal growth
- personal growth
- knowledge seeking
- personal growth
- personal gain
- self-improvement
- personal gain
- personal gain
- positive impact on life
- personal growth
- personal gain
- self-improvement
- self-worth
- self-esteem/worth
- personal growth
- personal growth
- personal growth
- learning
- learning
- personal growth
- self-improvement (mental health)
- personal growth
- personal growth
- personal growth
- personal growth
- learning via experience
- learning
- personal benefit/gain
- personal growth
- personal gain
- personal growth
- personal gain

- growth
- growth
- personal growth
- skills building
- personal gain

- comfortable role
- convenient
- fits with life
- fit with needs/preferences
- low commitment
- need to fit life
- need to fit life
- convenience
- convenient
- fits with life
- fits with life
- fits with schedule
- convenient
- convenience
- convenient
- fits with life
- needs to fit with schedule
- convenient
- convenient
- accessible
- fit with life/needs
- need to fit needs
- fits with life
- convenient
- fits with life
- fits with life
- convenient
- fits with life
- fit with lifestyle
- fits with life
- fits with life
- convenience
- convenient
- accessible
- convenience
- fits lifestyle
- convenience
- convenient
- accessible

- convenient
- fits with life
- accessibility
- fits with life
- convenient
- fits with life
- ease of application
- ease of access/application
- accessibility
- accessible
- accessible info
- accessibility

- comradery
- community
- comradery
- social engagement
- part of something
- social outlet
- social
- values social outlet
- social engagement
- social aspect
- community
- social outlet
- community
- community
- community integration
- social engagement
- belonging
- social outlet
- social outlet
- social outlet
- community
- social outlet
- social aspect is important
- social
- community
- social encouragers
- social outlet
- personal connections (e.g. friendships, closeness)
- social engagement
- social engagement
- community

- positive community
- positive community = important
- community
- community
- community
- social engagement
- personal connections
- community
- social engagement
- connection with clients
- comradery
- social engagement
- part of community
- personal connections
- volunteering = social?
- social growth
- positive social experiences
- social engagement
- community
- community
- social opportunities
- social opportunities
- community
- social opportunities
- community
- social opportunities
- social engagement
- social engagement
- positive social experiences
- comradery
- socialising
- socialising
- social engagement
- comradery
- social engagement
- community
- values social opportunities
- social engagement
- belonging
- community
- community
- social engagement
- genuine bonds
- friendship
- genuine bonds

- teamwork
- *volunteering for social reasons -> more friendship than professionalism*

- volunteer in absence of work
- work alternative
- volunteering isn’t work
- volunteering isn’t work
- more casual than work
- not work
- easy tasks
- work alternative
- more casual than work
- not as serious as work
- low stress
- low stress
- job alternative
- more structure than expected
- more structure than expected
- expected more casual
- low effort
- change from work
- volunteering is an extra
- enjoys volunteering more than work
- easier than working
- easier than job-seeking
- not a priority
- volunteering = extra
- volunteering seen as easy
- expected volunteering easier
- volunteering - preference due to low stress
- volunteering seen as low stress
- volunteering isn’t work
- alternative to work
- expected easier role
-

- poor previous role
- desire for change
- poor life circumstances

- prosocial action

- prosocial action
- prosocial action
- personal growth
- giving back
- Time to give
- helping others
- value for other people
- empathy
- empathy
- prosocial/altruistic
- helping others
- prosocial action
- prosocial action
- personal investment
- care
- care
- facilitate positive change
- help needy
- focus on others
- prosocial action
- care for clients
- helping needy
- value for clients
- empathy for clients
- personal investment/connection
- care/empathy
- empathy
- helping others
- prosocial action
- helping those in need is fulfilling
- empathising
- personal understanding
- prosocial/altruistic
- altruism
- help needy if can
- good deed
-

- applying existing skills
    - *could this go with useful/valued?*
- related to existing skills - (familiar work)

- worthwhile

- creates opportunity
- stepping stone to employment
- opportunity
- opportunities
- job opportunities
- employability
- experience for employment
- values opportunity

- family encouragement
- engaging/enjoyable
- social encouragement
- social encouragement
- family encouragement
- social encouragement
- social encouragement
- social engagement
- social engagement
- social encouragement
- social encouragement
- social encouragement

- choice of roles
- role variation
- diverse roles

Participant 8 - Victor

- building bonds
- client focus
- client autonomy
- client-focus
- adequate training
- forgotten
- value congruence between intention/action
- supported by org
- autonomy
- responsibility
- awareness of services

- social encouragement from others
- values enough to encouraging others
- sees volunteering as part of lifestyle
- diverse roles to suit needs
- values communication
- volunteer communication
- mutual learning
- communication
- communication is important
- clear client appreciation
- expect appreciation
- comfortability
- client-focussed
- care for clients
- uncomfortable tasks
- values punctuality
- organisation/time-management
- volunteer time not valued
- frustrated

Participant 9 - Charlie

- devalued due to age
- improved mindset/ QOL
- perseverance
- positive QOL/outcomes
- personal understanding
- challenging
- value autonomy
- communication with org
- values communication
- pension is motivation - convenient
- reduction of stress
- life uncertainty
- personal focus
- clients value service

Participant 10 - Louise

- reputable organisation
- willing to contribute lots of time
- makes good/fair decisions for volunteers
- aligned with values
- prosocial action
- values communication
- support
- variety in tasks

Participant 11 - Karen

- improve work ethic

Unsure if relevant (maybe not **motivation**)

- personal connection to clients
- longstanding connections
- structure restricts connection
- organisational change = changed focus
- personal connection with clients
- clients vs friends
- volunteers previously more valued
- growth = disconnect
- communication
- self-worth
- age
- change for better/worse?
- VTF different to other organisations
- adequate training
- hands on learning
- more learning
- sacrifice time
- age-related barriers
- prefer less structure
- client focussed work
- personalised
- more social
- company listens to needs/wants
- positive view of org
- client-focussed
- communication
- difficulty accessing
- communication
- communication breakdowns
- communication
- communication
- lost interest in role
- change if not suitable
- change if unsuitable
- personalised care
- more convenience
- barriers to application
- org. flexibility re change
- two way communication

- positive volunteer environment
- positive impact on mentality
- change, uncertainty
- client centred care
- communication
- barriers to care
- procedural barriers
- genuine bonds
- change
- restricted by rules
- client focussed care
- communication
- communication
- restricted by rules
- clients or closer
- good support = personalised
- perseverance
- organisation appreciation
- comfortable with staff
- open communication
- build own understanding
- required to volunteer
- positive impact for clients and volunteers
- older people volunteer
- retirement
- support
- family support
- social encouragement
- social encouragement (friends)
- social encouragement
- dynamic role
- support (family, volunteers, organisation)
- barriers to volunteering
- personalised support
- supported
- unexpected benefits
- supported
- barrier (less autonomy) - values autonomy
- personal drive
- easy application - facilitated
- support from others
- support
- improved QOL
- employment opportunities/raises employability
- mutual benefit

- support between staff - comradery
- support from staff
- barriers to helping
- uncertainty
- client-focussed
- care for clients
- client-focus
- client-focussed
- client-centred work
- role variety
- equipping to help clients
- mutual benefits

- positive environment
- supportive/positive environment
- supportive management
- listened to by org
- positive environment
- support system
- support in training
- support from org/management
- organisation hears volunteers
- input valued
- able to offer skills/insight
- felt listened to by org
- heard by organisation
- valued/listened to
- values good management/coordination
- support to continue
- importance of being heard
- valued by organisation
- positive volunteer environment
- organisation can facilitate community
- supported by org
- org. values time given
- supportive org
- support from org
- less clear appreciation from org
- focus shifted from volunteer recognition
- positive volunteer environment
- positive volunteer environment
- organisation listens
- support from org
- support valued

- support
- views organisation positively
- support
- positive volunteer environment
- support from org
- support from org
- support from org
- supported by org
- consistency in role
- support
- supported by org
- positive volunteer environment
- supported by organisation
- values support
- positive volunteer environment
- supportive environment
- not heard by org
- not valued throughout merger
- value from org = important
- no volunteer recognition
- not heard by org
- communication
- expect org to listen
- communication
- input
- support from training
- supportive organisation
- communication
- supported by organisation
- support from organisation
- supportive org
- org doing good for volunteers
- mutually beneficial
- positive view of org
- supported by org
- supportive org
- positive view of org
- organisation is equitable
- positive volunteer environment
- positive volunteer environment
- positive volunteer environment
- positive volunteering environment
- appreciates support/guidance
- appreciates guidance
- guidance

- positive volunteering environ
- support
- support
- positive volunteer environment
- support
- supported to continue role
- individualised support
- values good communication
- valued by organisation
- value organisation listening
- support from organisation
- supported by org
- support from organisation
- positive environment
- org supports volunteers
- supportive organisation
- approachable
- clear communication
- accepting and supportive environment
- support from organisation
- organisational support

Part 3 - Themes write up, preliminary

THEME 1: WHAT'S IMPORTANT TO ME?

- **Personal interest/enjoyment, variety (interesting), alignment with values**
- **"What's important to me?"**
    - Interviewees often mentioned that their roles encompassed either tasks they enjoyed or were interested in (e.g. gardening).
    - Spoke about aligning their roles with their values
    - Noted that role variety was an attractive part of volunteering, and so it appears that having a choice of tasks may retain interest.

The theme What's Important to Me? - importance of personal interest and role enjoyment expressed by interviewees when speaking about experiences.

- "I love gardening and I love talking to people and that seemed a good fit..."
- "I was trying to retire and do something interesting...".

Variety in volunteer roles and settings - way of keeping volunteering experiences dynamic and engaging.

- "...we've even discussed you know, like every fortnight we go to (***organisation name***) or something like that and then for the other weeks, we go to a different organisation and do something else. Just to give that sort of difference."
- "... you can choose to do like gardening now and you see someone doing something else like home shopping or all that, you can like ask the manager to like 'Can I try this for let's say a few weeks?' And then you can go and do that and if you find you like that a bit more than the gardening, they can maybe keep you on the home shopping..."

Volunteer role aligned with values:

- "I've always wanted to give back to the community. You know?"
- "And we were doing something good to help other people…". It seems that in these
    - values of altruism, wanting to give back to the community in a selfless way

Mental health benefits:

- "...that sort of put a big black could over my head. I think that doing what I'm doing now has really helped in that aspect as well.".

THEME 1.1: Finding Purpose

- aspirations to meaningfully engage in everyday life.
- keeping themselves busy
- staying active and out in the community
    - "...I decided that I wanted to retire and close up my business – everything and I needed something to do."
    - "...I don't feel like I'm just doing nothing. Even though maybe I'm not doing that much. It's better than doing nothing…".
    - Lack of activity = purposelessness

Purpose/usefulness derived from volunteering

- "I wanted to be associated with an organization which is actually contributing to the society..."

THEME 2: LEARNING AND GROWTH

- **Personal growth, skills building, gain, self-improvement, learning, - Learning and growth**
    - Positive appraisals of engaging in volunteering, learning opportunities
    - What is it that these volunteers seek to gain? "personal gain"
    - Learning new skills, building on experience
    - emotional/confidence benefits

Skills building - useful outside of volunteering

- "...it teaches me things that I can use myself here on my own property"
- "I got a certificate to use a chainsaw and quite a few other things. Which benefitted me."

Value-laden learnings. Less tangible, more intrinsic. e.g. confidence, self-efficacy.

- "I've learnt that I can do more than I thought I could."
- "I'm not someone that can just walk up to someone and start talking to them and I think the social interaction … with the volunteering, it's actually given me a new lease on life."

Learning as helpful for employment - career-related skills

- "...it can also open up doors for employment later on. If you have liked worked long enough and the general going out there and meeting people and working with a team..."

THEME 3: SOMEWHERE TO BELONG

- **Comradery, social engagement, sense of community, belonging**
    - It appeared that opportunities to meet new people and engage socially were important elements of volunteering.
    - Seek to meet new people, engage socially, and be a part of a broader community
    - Looking for belonging
    - Some volunteers spoke to the notion of being a part of a broader community, or finding a sense of belonging within a setting such as a volunteer organisation.
    - Like-minded, teamwork
    - **motivation to stay
    - Organisation hearing desires expressed by volunteers /
    - feedback that social events foster warmth and connection in volunteering
    - create warmth and community
    - listen to volunteers

Initial reasons for volunteering, sense of community, meeting others.

- "I also wanted to participate in the community was the main reason. Just be part of the community I lived in and to help people.".
- "I'm starting to go to social. I would recommend it because if they loved going out and meeting new people or doing stuff or if they just want to like join a group, it's nice to have people that you can talk to.".
- "I do like the volunteering thing and it's kind of nice to get out and it helps to meet people and like-minded people"

Opportunities for socialisation via volunteering

- "…all the Depots used to get together and we'd have a central breakfast in a big hall or something. Then it got down to – we'd just have a get-together in May of every Depot.".
- "Even if it's only an hour or one day a month. The hardest thing is trying to get everybody together. But I don't think that should stop it happening.".

THEME 4: (Meet me in the middle?)

- **Convenience, fit with life/lifestyle, accessible info & locations**
    - Interviewees appear to consider whether volunteering roles fit with their lifestyle,
    - More about how the org can fit with what the volunteers have to offer
    - The convenience of the volunteer role appears to be important to interviewees - e.g. distance, available times, work in around other aspects of life
    - Volunteering is secondary/auxiliary???

The accessibility of volunteering settings and opportunities seems to be a salient element in (how willing interviewees were to volunteer) (how easy taking part in volunteering)

- Or rather, is it that volunteering is a sacrifice, and so it should be easy to find a role?
- Location (geographical access, ease of travel)
- Fit with schedule (times available) -
    - sense that volunteers are literally volunteering their time, so perhaps organisations should accommodate this by offering many times that fit
    - volunteering given the convenience - volunteering isn't limiting to any other part of their life. rather, fulfilling. i.e. volunteering fits with the other elements of the interviewee's lifestyle
- Others pointed out gaps in accessibility - such as information access pre-volunteering

Location, accessability

- "...it's sort of 'local' to me as well, the Depot for gardening. I can walk there and also I can run if I need to get there quickly…".
- "...if they moved from (***location 1)*** to (***location 2***) or somewhere. You know, that's time to get there and whereas it's convenient for me to volunteer where I'm volunteering now.".
- "The only challenge that I had personally was because I don't drive, I found it a bit of challenge to get from where I'm living to the venue - to get from the venue and to get home again. Because although it's on bus route, it's in not a very nice area."
- *"Although he never objected and I just didn't feel that I was doing the right thing. So I thought 'Well, I'll have to leave this then and find something else to do.' Which I did reluctantly"*

Flexibility aids in accessibility

- "...they're flexible. Like I didn't have to commit to being there every week or something. Like I've got other things that I do. I can't always do it because I've got grandkids.".

THEME 5: Not just a number

- Fulfilment, feeling valued, recognition, appreciation
  - Feeling heard by org, having their needs met
  - Feeling like their input is appreciated by the organisation and consumers
  - Enjoy the recognition and appreciation which [is assumed to] (- *is it?*) come with volunteering
  - Stay due to feeling appreciated & seeing differences made in consumer's lives - understand they're valued by the people who they provide services to

Support volunteers feel from organisation, opportunity to have voice.

- "The supervision we get from the organization is good. The co-ordinators that we work with are very good. Yeah. It's good in that way."
- "They're always asking you 'Are you sure you can do that' or you know, 'Do you want a hand with that or' – now they're always very protective of their volunteers."
- "She spoke to her manager and they organised for the HACC taxi to actually bring (*name redacted*"

Communication between volunteer and organisation as a form of support

- "...just even a text like on the day. Like not me just having to call up. If they'd just say 'Oh, you know,' because literally all I'd do is say 'What run are we going to be on tomorrow?' and that's it. Like they could literally just send me a text and then I'd know because I spoke to them."

Positive recognition from organisation AND consumers as a form of support and appreciation, validation of efforts. Enjoyment.

- "They celebrate the volunteers and they have volunteer awards."
- "They will have a Christmas picnic. All those little things to say 'Thank you' and it's something that is really good and to know that you are appreciated. You know, from the organisation.".
- "...that does make you feel really good. When they appreciate what you do."
- "volunteering is giving your time when you don't get paid. You know? No, I enjoy it and we have support, because the volunteers are appreciated."
